# Large‐scale assessment of intra‐ and inter‐annual breeding success using a remote camera network

**DOI:** 10.1002/rse2.171

**Published:** 2020-08-31

**Authors:** Casey Youngflesh, Fiona M. Jones, Heather J. Lynch, Joan Arthur, Zuzana Ročkaiová, Holly R. Torsey, Tom Hart

**Affiliations:** ^1^ Department of Ecology and Evolutionary Biology University of California Los Angeles CA 90095 USA; ^2^ Department of Zoology University of Oxford Oxford OX1 3SZ UK; ^3^ Institute for Advanced Computational Science Stony Brook University Stony Brook NY 11794 USA; ^4^ Zooniverse Department of Physics University of Oxford Oxford OX1 3RH UK

**Keywords:** Capture‐recapture model, global change, remote cameras, seabird, time‐lapse cameras, breeding success

## Abstract

Changes in the physical environment along the Antarctic Peninsula have been among the most rapid anywhere on the planet. In concert with environmental change, the potential for direct human disturbance resulting from tourism, scientific programs, and commercial fisheries continues to rise in the region. While seabirds, such as the gentoo penguin *Pygoscelis papua*, are commonly used to assess the impact of these disturbances on natural systems, research efforts are often hampered by limited spatial coverage and lack of temporal resolution. Using a large‐scale remote time‐lapse camera network and a modeling framework adapted from capture‐recapture studies, we assess drivers of intra‐ and inter‐annual dynamics in gentoo penguin breeding success across nearly the entire species’ range in the Atlantic sector of the Southern Ocean. We quantify the precise timing of egg/chick mortality within each season and examine the role of precipitation events, tourism visitation, and fishing activity for Antarctic krill *Euphausia superba* (a principal prey resource in the Antarctic) in these processes. We find that nest failure rates are higher in the egg than the chick stage and that neither krill fishing nor tourism visitation had a strong effect on gentoo penguin breeding success. While precipitation events had, on average, little effect on nest mortality, results suggest that extreme weather events can precipitate sharp increases in nest failure. This study highlights the importance of continuous ecosystem monitoring, facilitated here by remote time‐lapse cameras, in understanding ecological responses to environmental stressors, particularly with regard to the timing of events such as extreme weather.

## Graphical abstract

The Antarctic Peninsula is currently experiencing rapid environmental change as well as direct anthropogenic pressures. Using a remote time‐lapse camera network coupled with a novel modeling framework, we quantify the intra‐ and inter‐annual responses of gentoo penguin breeding success to these pressures. While we find little effect of average conditions on demographics, extreme environmental events can precipitate sharp increases in mortality, highlighting the importance of intra‐annual dynamics and non‐linear relationships in demographic processes.

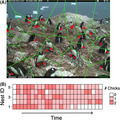

## Introduction

The Antarctic Peninsula (AP) region is currently experiencing rapid change resulting from both direct and indirect anthropogenic impacts. Air temperatures have increased significantly over the recent past (Mayewski et al. 2009), coinciding with changes in sea ice dynamics (Stammerjohn et al. 2012) and precipitation (Turner et al. 2005; Kirchgäßner 2011). Tourism visitation (Bender et al. 2016) and commercial fishing efforts (Nicol et al. 2012) also continue to increase in the region.

Given the complexity of natural systems, assessing the repercussions of these various stressors on Antarctic ecosystem dynamics is a difficult task. Seabirds, such as the gentoo penguin *Pygoscelis papua,* are commonly used to assess the impacts of environmental and anthropogenic stressors, as these birds are easily monitored and represent key predators in marine ecosystems. While gentoo penguins are often considered ‘climate change winners’ in the AP region (Hinke et al. 2007; Clucas et al. 2014), it is uncertain how intensifying anthropogenic stressors and abiotic changes are influencing populations. Other penguin species, such as the Adélie penguin *P. adeliae*, have shown marked declines in the AP region in recent years (Lynch et al. 2008, 2012). Disentangling how demographic processes in the *Pygoscelis* spp. penguins are responding to various threats driving population changes is key to understanding how the Antarctic system is changing over time and forecasting how these species might fare into the future.

The increasing frequency of precipitation events observed in the AP region (Turner et al. 2005; Kirchgäßner 2011) is a key concern for penguins on the western Antarctic Peninsula, particularly with regard to breeding success and chick survival. Nest flooding, resulting from heavy rainfall or accumulated snowmelt (Youngflesh 2018b), may lead to substantial heat loss in chicks and provides one mechanism for the negative relationship observed between precipitation and breeding success for *Pygoscelis* spp. and Magellanic (*Spheniscus magellanicus*) penguins (e.g. Lynch et al. 2010; Boersma and Rebstock 2014; Ropert‐Coudert et al. 2015). This can impact survival at both the egg and chick stages (Massom et al. 2006; Thyen and Becker 2006; Cimino et al. 2014). Model results suggest that wetting of just 10% of the total chick surface area is sufficient to reduce survival probability through decreased fledging mass (Lustick and Adams 1977; Konarzewski and Taylor 1989; Chapman et al. 2011). Increased precipitation has been associated with reduced chick growth rates in a number of bird species (e.g. Dunn 1975; Konarzewski and Taylor 1989; Thyen and Becker 2006), an effect owing to associated heat loss due to wetting and/or a continued decrease in provisioning rate owing to adverse weather conditions (Moss 1979).

Direct anthropogenic threats, such as an expanding Antarctic krill *Euphausia superba* fishery, are also cause for concern (Pfeiffer and Peter 2004; Nicol et al. 2012). Krill, an important food resource for *Pygoscelis* spp. penguins (Volkman et al. 1980), currently represent the largest (by tonnage) fishery in the Southern Ocean, with a catch limit of 620 000 tons per year (Commission for the Conservation of Antarctic Marine Living Resources [CCAMLR], 2018). It is important to understand how krill harvest may be impacting predator populations in the Southern Ocean, particularly in the southwest Atlantic sector (20°W–80°W), which has historically been the primary focus of the industry (Kawaguchi et al. 2009). An increase in demand for krill‐derived products, combined with the potential for a spatial expansion of fishing as sea ice cover declines, further heightens the need for a more complete understanding of the impact of this threat (Kawaguchi et al. 2009; Nicol et al. 2012; Stammerjohn et al. 2012).

In addition to precipitation and the removal of key prey, tourism‐related impacts are also a concern for gentoo penguins. The tourism industry in Antarctica has grown rapidly in the last several decades, with more than 58 000 visitors during the 2017/2018 season (Lynch et al. 2019). More than 70% of all tour ship landings during the 2012/2013 austral summer occurred at gentoo penguin colonies (Bender et al. 2016). Disturbance of breeding individuals at colonies is the primary concern, as visitation of colonies may have physiological impacts (e.g. stress) or present opportunities for predators to prey upon eggs or young chicks. Prior studies examining the impact of site visitation on gentoo penguins, however, have drawn inconsistent conclusions (Holmes 2007; Lynch et al. 2019).

Effectively evaluating the impacts of these potential threats on gentoo breeding success requires a substantial monitoring effort. Historically, assessing demographic parameters such as egg and chick survival on a large spatial scale with high temporal resolution has been both challenging (logistically and financially) and labor‐intensive, resulting in a trade‐off between the depth of demographic information and geographic breadth (Lynch et al. 2012). Remote time‐lapse cameras, pioneered for use in penguin monitoring studies by Newbery and Southwell (2009), provide a solution to this problem, allowing colonies to be observed at high frequency across large spatial extents (Hinke et al. 2018; Jones et al. 2018). As such, they facilitate the quantification and comparison of breeding success across large regions and provide an ongoing opportunity to quantify the timing of mortality events across the breeding season. Here we use data from the large‐scale *Penguin Watch* remote time‐lapse camera network (Jones et al. 2018) to examine breeding success at 14 gentoo penguin colonies across the AP region and South Georgia (representing the bulk of the species’ South Atlantic sector range), providing the most spatially comprehensive study of gentoo demographics to date. We employ a modeling framework adapted from capture‐recapture studies to explicitly account for non‐detection (owing to obscuration of chicks by adults, poor visibility, or human error), and assess changes in chick survival throughout the breeding season. Our goals were to assess: (1) large‐scale patterns of gentoo penguin breeding success across a large portion of their breeding range, (2) variation in the timing of chick mortality over the course of the breeding season, and (3) the impact of three hypothesized drivers of breeding success: precipitation, tourism visitation, and commercial krill fishing.

## Materials and Methods

### Image data collection via the *Penguin Watch* remote time‐lapse camera network

Images were captured by Reconyx HC500 Hyperfire Trail Cameras (commercially available; Reconyx Inc., Holmen, WI, USA), deployed according to the methodology outlined by Jones et al. (2018). These devices form part of the *Penguin Watch* remote time‐lapse camera network, which currently comprises approximately 100 units positioned across the Scotia Arc region (Jones et al. 2018). Devices were programmed to capture images once per hour throughout the breeding season, which were stored on Secure Digital (SD) cards and retrieved the following austral summer.

### Chick classification from remote time‐lapse camera images

**Figure 1:**
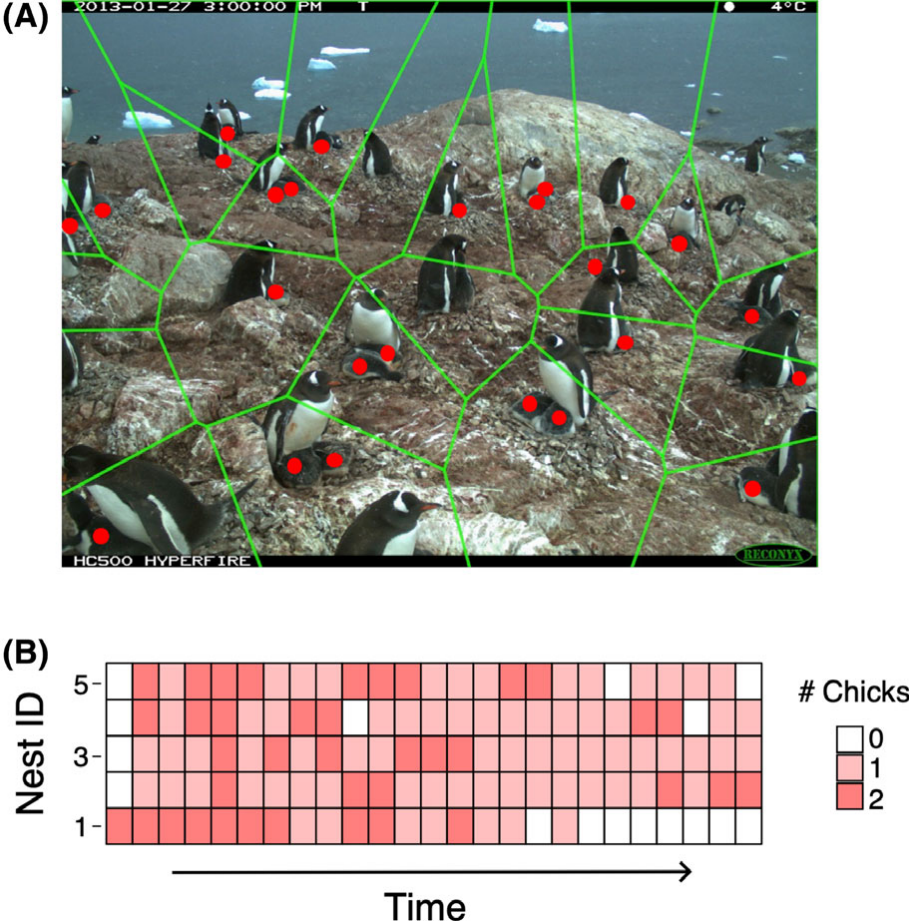
(A) Image of a penguin nesting site obtained from the *Penguin Watch* remote time‐lapse camera network. Red dots represent penguin chicks (classified by authors using the *Penguin Watch* platform on Zooniverse); green polygons represent nest zones (delineated using a Voronoi tessellation with nest locations as ‘seeds’); (B) sample time series of the number of chicks observed in each of five hypothetical nests. At each time step zero, one, or two chicks could be observed.

Penguin chick survival was assessed using images captured at 14 gentoo penguin colonies across the AP and South Georgia between the 2012–2013 and 2017–2018 breeding seasons (approximately December – February; total sites/years = 28; full list of data availability given in Appendix S1, Table S1‐1). Cameras captured images every hour during a set daytime period (for example, 0500 to 2100), with an average of 16 images taken per day. The locations of individual nests were annotated on a representative image from each site/year, which were then used to demarcate ‘nest zones’ through Voronoi tessellation using the package ‘deldir’ (Turner 2019) in the R statistical environment (R Core Team^2019^, Fig. 1A, Appendix S1). Voronoi tessellation partitions space into polygons with one ‘seed’ (nest location, in this case) per polygon, where all possible points inside that polygon are closer to that seed than any other seed.

These zones remained constant throughout each site/year image set, as the camera positions were fixed. The R packages ‘jpeg’ (Urbanek 2014) and ‘stringr’ (Wickham 2019) were used to render and manipulate images obtained from the remote time‐lapse camera network and to support data processing, respectively. Images were annotated using a version of the online citizen science project *Penguin Watch* (www.penguinwatch.org) specifically designed for the purposes of this study (i.e. not publicly accessible). Using this interface, we classified the location of penguin chicks in each image (total number of images = 9831). We defined our focal period as the time between the sighting of the first chick and the crèche stage (the period during which penguin chicks begin to form large chick aggregations). All identified nests were included in the analysis, irrespective of survival outcome so as not to bias survival estimates (i.e. nests where no chicks hatched were included). As gentoo penguins consistently lay two eggs per clutch, each nest could have zero, one, or two chicks present in each image. Image classifications were used to create time series of the observed number of chicks in each nest zone for each site/year (Fig. 1B). These time series were used in a modified capture‐recapture framework (Nichols 1992; where each observation represents a ‘capture’ for a particular nest) to assess how chick mortality changes throughout a season, and to what degree changes in chick mortality coincide with weather events. While traditional capture‐recapture studies rely on marking individuals, the locations of the nests serve as distinct markers to reference the identity of each chick/sibling pair.

### Modeling chick survival in a modified capture‐recapture framework

Given the possibility of detection failure, the observed state of a penguin chick in any given image (present/absent) cannot be assumed to be the true state (truly present/truly absent). A Bayesian state‐space framework was implemented to model both the true (*z*) and observed (*y*) states of penguin chicks at each time step (*t*), for each nest (*i*), year (*j*), and site (*k*). Hourly data were aggregated to daily resolution (i.e. the highest number of chicks seen in a given nest over the course of a day) for computational reasons. The Bayesian approach provides a flexible modeling framework and allows for the propagation and quantification of uncertainty throughout the analysis (Gelman and Hill 2006). Crèche dates for each site/year were used to determine an approximate lay date (60 days before crèche date; Hinke et al. 2012). This extrapolated lay date was used as the initial time step of the model so that time series for each site/year were 60 time steps (days) in length. Each nest was assumed to have two eggs at the initial time step, as this is highly conserved within the *Pygoscelis* spp. penguins (Borboroglu and Boersma 2013).

The true state (alive/dead) of each chick in a given nest depends on its state in the previous time step. This is a binomial process, with daily survival probability ϕ, which was modeled as a function of a global intercept term ( $\mu_{\phi }$) that varies by site and year. As the two chicks in a given nest cannot be uniquely distinguished in an image, the survival of both chicks was modeled jointly with a binomial distribution (as opposed to the Bernoulli models typically used in capture‐recapture studies). This is given as,

(1) $$z_{t,i,j,k}∼\text{Binom}\left(z_{t-1,i,j,k},\phi_{j,k}\right)$$

$$\text{logit}\left(\phi_{j,k}\right)=\mu_{\phi_{j,k}}$$

where *t* is the time step for nest *i* in year *j* at site *k*. $\mu_{\phi_{j,k}}$ represents the (logit‐transformed) survival of each egg/chick in year *j* at site *k*. A logit transform was used to more easily set priors for this parameter. We assumed that site‐ and year‐specific survival *ϕ* (logit transformed) follows a Normal distribution, which allowed for random variation in the site‐ and year‐specific survival parameters under the constraint of a common distribution,

(2) $$\mu_{\phi_{j,k}}∼N\left(\theta_{\phi },\sigma_{\mu_{\phi }}\right)$$

where *θ_ϕ_* and $\sigma_{\mu_{\phi }}$ represent the grand mean and standard deviation for this parameter, respectively, with larger variation among sites and years reflected in larger estimates for $\sigma_{\mu_{\phi }}$.

Unavoidably, camera trap images provide information only on what is visible. The contents of a nest may be obscured by the parent or artifacts caused by rain on the camera lens (Southwell and Emmerson 2015). It is therefore necessary to model non‐detection of nest contents, which provides a bridge between what actually exists in each nest and what can be observed. The observed state of each nest (*y*) was similarly modeled as a binomial process, with detection (*p*), that depends on the true state (*z*) of that nest in that time step,

(3) $$y_{t,i,j,k}∼\text{Binom}\left(z_{t,i,j,k},p_{t,i,j,k}\right).$$

Since chicks were the quantity of interest in this study and egg sightings were not quantified, data were only included for *y* over the period of interest for each site/year, from the first sighting of a chick to the start of the crèche period (that is, NA values were used prior to the sighting of the first chick for a given site/year). The logit of the detection probability for time step *t*, nest *i*, year *j*, at site *k* (*p_t,i,j,k_*) was modeled as a function of a global intercept ( $\mu_{p}$), a time‐varying parameter (*β_p_*) that varied by year and site to account for the change in detection probability of a penguin chick as it grows larger over time (*x*), and an offset for each nest, year, and site ( $\nu_{p}$), as detection is likely to differ across nests, sites, and years depending on the position of the camera and nest,

(4) $$\text{logit}\left(p_{t,i,j,k}\right)=\mu_{p}+\nu_{p_{i,j,k}}+\beta_{p_{j,k}}x_{t}$$

In this way, detection probability can vary across time, nest, site, and year. Note that while *β_p_* represents a linear function of time in logit space, this corresponds to a logistic function in probability space (as illustrated in Appendix S1, Figs. S1–S5). $\mu_{p}$ and $\nu_{p}$ impact where the inflection point of the detection curve is located, while *β_p_* impacts the shape of the detection curve. Both *β_p_* and $\nu_{p}$ were modeled hierarchically, with means $\mu_{\beta_{p}}$ and 0, and standard deviations $\sigma_{\beta_{p}}$ and $\sigma_{v_{p}}$, respectively,

(5) $$\beta_{p_{j,k}}∼N\left(\mu_{\beta_{p}},\sigma_{\beta_{p}}\right)$$

$$\nu_{p_{i,j,k}}∼N\left(0,\sigma_{\nu_{p}}\right).$$

The total number of chicks at each site *k* in every year *j* at every time step *t* (*Z_t,j,k_*) was calculated as a derived quantity, by summing across the number of chicks alive (*z_t,i,j,k_*) at each nest *i*,

(6) $$z_{t,j,k}=\sum_{i}z_{t,i,j,k}.$$

Breeding success (chicks per pair) for each site/year was calculated as a derived quantity, by dividing *Z*_60,_ *_j,k_* (the total number of chicks alive at the start of the crèche period) by the total number of nests at each iteration of the posterior chain. Models were fit using the R package ‘rjags’ (Plummer 2016) to interface with JAGS (Plummer 2003) in the R statistical environment (R Core Team 2019). Inferences were obtained from 400 000 samples drawn from six chains with a thinning rate of 100, following a ‘burn‐in’ period of 400 000 draws and an adaptation phase of 8000 draws. Convergence of the model was assessed via a visual analysis of the posterior chains, in addition to the use of the Gelman‐Rubin convergence diagnostic (Brooks and Gelman 1998). Models unambiguously converged (Rhat < 1.1) and effective sample sizes were sufficiently large (ess/chain > 100). Posterior predictive checks (Gelman et al. 2013), whereby data generated by the model are compared to the observed data, did not indicate any model misfit (Appendix S1). The R packages ‘MCMCvis’ (Youngflesh 2018a), ‘dplyr’ (Wickham et al. 2018), and ‘ggplot2’ (Wickham 2016) were used to manipulate data and model output, and to create all plots.

### Quantifying changes in chick mortality over the breeding season

The estimated number of chicks at each time step (*Z_t,j,k_*) was used to determine how rates of mortality varied across the breeding season. The modeled breeding season was divided into three segments. The first 30 days were classified as the ‘egg period’, the next 15 days were classified as the ‘young chick period’, and the final 15 days were classified as the ‘old chick period’. While these periods were chosen somewhat arbitrarily, they represent early, middle, and late stages of the egg/chick portion of the breeding season. To calculate the average daily mortality over each of these periods, for each site/year the difference between the estimated number of chicks at the beginning and end of each period was divided by the total number of days for that period (i.e. the change in number of chicks per day). Average daily mortality for each of these periods was aggregated across sites and used to create density plots (Fig. 3). Differences in mortality rates across these periods were assessed using a Friedman test, used to test for differences in means across repeated measures (measures represented by mortality rates for each site/year) without the assumption of normality (Friedman 1937).

### The effect of precipitation, fishing, and tourism on gentoo penguin breeding success

Each time‐lapse image was scored according to a precipitation scale by two authors independently, with any discrepancies resolved via discussion. The magnitude of rain events (observed as pooling on the ground) was scored on a scale from R1 (light rain) to R3 (heavy rain). Snow events were similarly classified, with S1 representing light snowfall with patchy coverage between nests, and S3 representing heavy snowfall (Appendix S1). One score was recorded per precipitation event – that is, if snow remained for several days before melting, only the initial, causative snowfall event was recorded (although any additional precipitation during this period was scored). The timing of precipitation events was visually compared to demographic dynamics within each season. The total number of precipitation events greater than or equal to magnitude 2 (i.e. R2, R3, S2, S3) was calculated for each site to investigate the effect of the total seasonal precipitation on breeding success across sites/years.

Monthly krill fishery catch data aggregated to CCAMLR small‐scale management units (SSMU; areas designed to aid in the management of the Antarctic krill fishery; Appendix S2) were obtained from the CCAMLR statistical bulletin (CCAMLR 2019). For a given month, krill catch within a 150km radius of each site was determined by calculating a weighted average of SSMU catch (where weights were determined by the proportion of total buffer area covered by a given SSMU; Appendix S2). The sum of the weighted average krill catch from March (of year *t*‐1) through January (year *t*) was calculated for each site/year (where *t* is the year in which a given breeding season ends). This metric represents all krill caught at each site prior to and during the breeding season. As gentoo penguins generally overwinter near breeding sites (Hinke et al. 2007; Black et al. 2017), this period would account for any carry‐over effects that fisheries may have on breeding success the following season (e.g. reduced adult body condition resulting from increased fishery activity which may lead to lower breeding success). A 150km buffer was used to account for the overwinter movement of penguins around the breeding site as well as the spatial advection of krill during the six‐month period. The R packages ‘rgdal’ (Bivand et al. 2019), ‘rgeos’ (Bivand and Rundel 2019), ‘raster’ (Hijmans 2019), and ‘sp’ (Bivand et al. 2008) were used to process all spatial data.

The number of tourist visits at each AP and South Georgia site in each year was determined from visitation data provided by the International Association of Antarctica Tour Operators (IAATO) and the Government of South Georgia and the South Sandwich Islands (GSGSSI), respectively. Visitation only over the period from the extrapolated lay date to crèche date was used, as we hypothesize this would have the most direct impact on nesting penguins.

Breeding success at each site/year was modeled as a linear function of the total number of precipitation events, log(total krill catch), and total number of visitors. Three separate models were fit to evaluate the effects of the hypothesized drivers on breeding success while accounting for uncertainty in estimates of breeding success. Breeding success was modeled as a linear function of the proposed driver,

(7) $${\text{bs}}_{{\text{obs}}_{i}}∼N\left({\text{bs}}_{{\text{true}}_{i}},\sigma_{{\text{bs}}_{i}}\right)$$

$${\text{bs}}_{{\text{true}}_{i}}∼N\left(\mu_{i},\sigma \right)$$

$$\mu_{i}=\alpha +\beta x_{i}$$

where bs_obs_ is the estimated breeding success (posterior mean of the derived quantity from the capture‐recapture model) for each site/year, *σ*_bs_ is the uncertainty about the estimate of breeding success for each site/year (posterior standard deviation of the derived quantity; this is given rather than estimated by the model), bs_true_ is the latent true state of breeding success, *x* is the covariate of interest for site/year *i*, *α* is the intercept parameter, *β* is the slope parameter, and *σ* is the residual error term. Models were fit using the R package ‘rjags’ (Plummer 2016) to interface with JAGS (Plummer 2003) in the R statistical environment (R Core Team 2019). Inferences were obtained from 10 000 samples drawn from four chains, following a ‘burn‐in’ period of 10 000 draws and an adaptation phase of 5000 draws. Models unambiguously converged (Rhat < 1.1) and effective samples sizes were sufficiently large (ess/chain > 100). Code to reproduce all analyses can be found on GitHub (https://github.com/Penguin‐Watch/penguin_watch_model).

## Results

**Figure 2:**
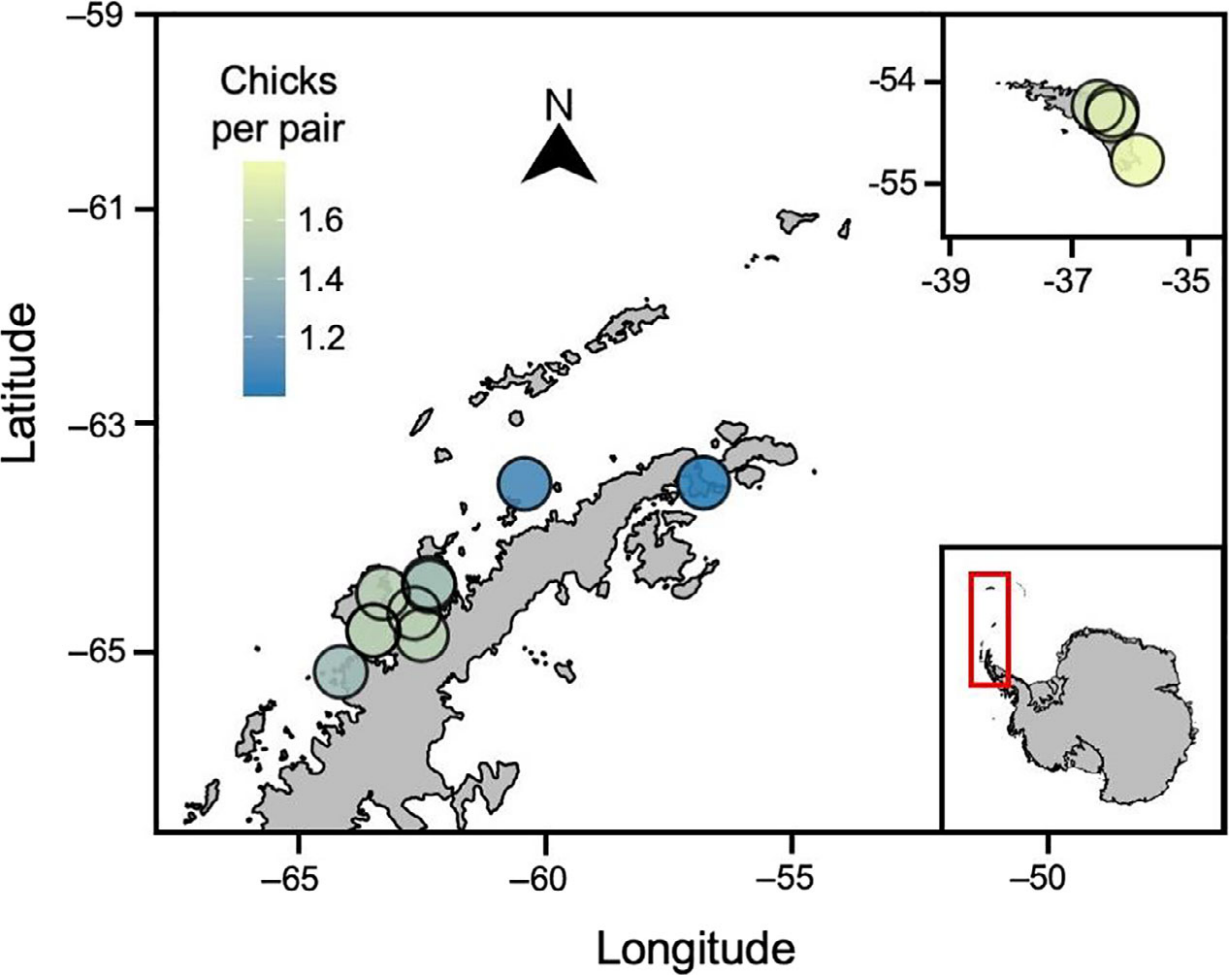
Average breeding success (number of chicks at crèche per nest averaged over all available years) at gentoo penguin colonies on the Antarctic Peninsula and South Georgia (inset). Yellow hues represent higher average breeding success, while bluer hues represent lower average breeding success for that site.

**Figure 3:**
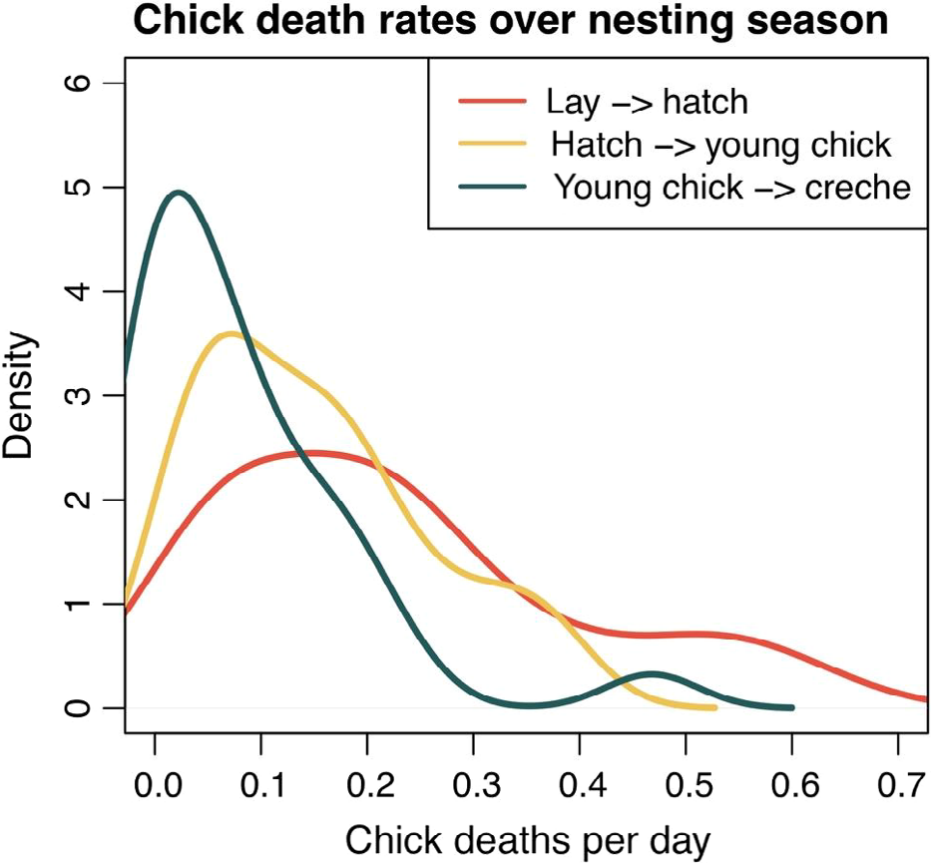
Density plots representing chick mortality in the ‘egg’ (red line), ‘young chick’ (yellow line), and ‘old chick’ (blue line) stages for each site/year. Mortality is higher during the earlier portion of the breeding season.

**Figure 4:**
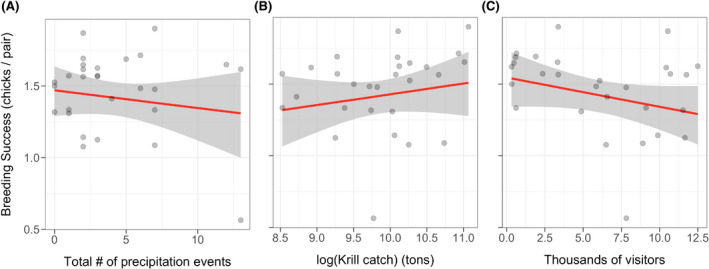
(A) Breeding success as a function of the total number of precipitation events, (B) breeding success as a function of log(krill catch), and (C) breeding success as a function of the total number of tourist visitors over the course of the breeding season. Each point represents a unique site/year, the red lines represent the linear model fit, while the gray ribbons represent the 95% credible intervals.

**Figure 5:**
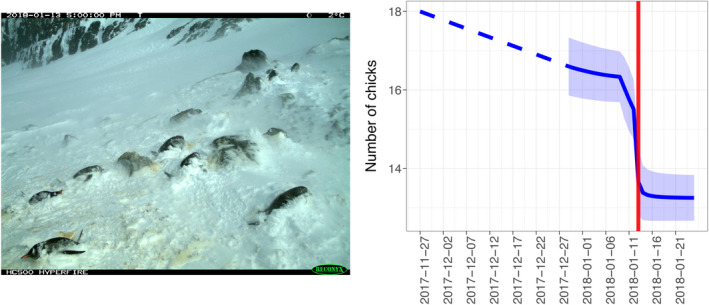
Left – image from the *Penguin Watch* camera network on January 13th, 2018, showing heavy snow covering penguin nests following a large storm at Brown Bluff (63.53° S, 56.89° W). See Video S1 for time‐lapse animation. Right – total number of chicks at this site over the course of the 2017–2018 breeding season. The solid blue line shows the estimated estimated total number of penguin chicks at each time step (*Z*) at this site/year. Uncertainty about that number (one standard deviation) is represented by the blue ribbon. The blue dashed lined represents the transition from the extrapolated egg lay date (60 days before crèche, where the number of chicks present is twice the total number of nests), to the date the first penguin chick was recorded in an image. The red vertical line coincides with the day the image shown at left was recorded.

Average breeding success varied across the spatial range of the study (Fig. 2; Appendix S1). Breeding success ranged from 0.56 to 1.90 chicks per pair and averaged 1.46 chicks per pair across all sites/years (Appendix S1). We found that chick mortality was higher earlier in the season (*P* < 0.001; i.e. during the egg period, rather than the chick period; Fig. 3). We found no strong effect of total number of precipitation events (Fig. 4a), no strong effect of krill catch (Fig. 4b), and no strong effect of tourism visitation (Fig. 4c) on penguin breeding success. Credible intervals overlapped zero in all three cases – median ( $\beta_{\text{precipitation}}$) = −0.01 [95% CI: −0.04–0.02], median (*β*_krill_) = 0.08 [95% CI: −0.09–0.24], median (*β*_tourism_) = −0.02 [95% CI: −0.05–0.01]. In general, the timing of precipitation events were not associated with declines in chick mortality (Appendix S1). Large snow events, however, coincided with a drop in the numbers of chicks (Fig. 5, Video S1).

## Discussion

This non‐invasive capture‐recapture framework, facilitated by remote time‐lapse cameras, provides a novel way of quantifying both the intra‐ and inter‐annual dynamics of breeding success. This study provides an important advance in our understanding of environmental and anthropogenic impacts on gentoo penguin breeding success and illustrates the importance of continuous monitoring in understanding ecological responses to stressors that result from global change. We show that neither moderate precipitation events, krill fishing pressure, nor tourism visitation strongly drive gentoo penguin reproductive dynamics at these sites, despite prior concerns regarding these proposed drivers. However, discrete extreme weather events had a disproportionately large impact in this regard.

### Timing of chick mortality events and the impact of precipitation

Mortality was found to be highest during the egg stage (i.e. the first 30 days of the breeding period), in agreement with prior findings (Hinke et al. 2012). This early mortality is, by definition, not linked to chick provisioning rate, though food availability could influence egg attendance (Eikenaar et al. 2003). While the overall number of precipitation events does not seem to have an effect on breeding success (Fig. 4A), there appears to be a non‐linear relationship between these two variables, where extreme weather events may have a disproportionately large effect on breeding success (Fig. 5), though the inherent rarity of these events makes it difficult to apply formal tests in this regard. Since it is only these large precipitation events which appear to have an effect on egg/chick survival, it is likely that nest flooding or chick wetting and subsequent hypothermia is the primary cause of mortality (Lustick and Adams 1977; Massom et al. 2006; Thyen and Becker 2006; Cimino et al. 2014). Rock (AP) and peat substrate (South Georgia) that make up gentoo penguin nests allow for drainage of precipitation, which presumably prevents flooding during low to moderate precipitation events, and hence may explain why these events do not appear to depress breeding success. During heavy snow events (such as those observed in this study, where snow may fall directly into, or be blown into, nests), the likelihood of egg/chick wetting or drowning is heightened. Whether the increased drainage potential of raised tussock/peat nests used by gentoo penguins in South Georgia is partially responsible for higher breeding success in this region compared to the AP (Fig. 2) is a subject that merits further study. An increasing frequency of precipitation events on the AP has been documented over recent years (e.g. Kirchgäßner 2011), and is predicted to increase under climate change projections (Uotila et al. 2007), which may result in lower breeding success of gentoo and other penguin species (e.g. Boersma and Rebstock 2014) in the future. Gentoo penguins are known to lay second clutches if the first fails early in the season (Mauget et al. 1995). This was observed at one site during a particularly heavy snow year over the course of this study (Brown Bluff [63.53° S, 56.89° W] 2015–2016), in addition to observations made prior to this study (Yankee Harbor [62.53° S, 59.78° W] 2007–2008). It is possible that instances of re‐laying will become more common as precipitation events increase in frequency, particularly if these extreme events occur early in the season.

### Effect of direct anthropogenic impacts on gentoo penguin breeding success

There has been much concern about the impact of krill extraction on populations of Antarctic predators (e.g. Croxall et al. 1999). Previous reports (CCAMLR 2003) have highlighted the difficulty of parsing the effects of commercial harvesting and environmental change using the existing CCAMLR Ecosystem Monitoring Program (CEMP) design. Here we present a new and unique dataset, to examine penguin demographics on unprecedented spatial and temporal scales. Future studies taking into account the spatio‐temporal scales on which krill and their predators (including fishing vessels) interact are necessary to determine whether catch limits are truly precautionary (Watters et al. 2020), and may be facilitated by the release of higher spatial‐resolution krill catch data in the future.

Prior studies have found overlap between penguin foraging areas and krill fishing zones (Hinke et al. 2017). For the gentoo penguin, however, krill fishing effort does not currently seem to be a major factor in driving breeding success at the sites included in this study (Fig. 4B). We also examined krill catch solely during the breeding season but found a similar lack of relationship (Appendix S2). This could be due to the dietary flexibility of gentoo penguins (Lescroel et al. 2004; Miller et al. 2009; Polito et al. 2015) and their near‐shore, on‐shelf foraging strategy (Ratcliffe et al. 2018), which is in contrast to the chinstrap penguin *Pygoscelis antarcticus* and Adélie penguin, though krill availability likely impacts other gentoo prey species (Nemoto et al. 1988). Krill fishing may have more of an impact during the overwinter period, particularly since harvesting rates are highest during this time (Appendix S2). This may influence the survival rates of juveniles, though these dynamics are outside the scope of this study. Finally, it is also possible that periods of higher krill catch simply reflect periods of higher krill availability (suggested by the weakly positive relationship between penguin breeding success and krill catch; Fig 4B), for fishing vessels and penguins, making it difficult to infer the true impact of krill fishing on penguin breeding success.

We also found no evidence that tourism visitation had an impact on gentoo penguin breeding success (Fig. 4C), consistent with recent findings that tourism had little relationship to hormonal stress markers in gentoo penguins on the AP (Lynch et al. 2019). This is in contrast to findings by Lynch et al. (2010) who documented an association between decreased gentoo breeding success and ‘frequent’ site visitation at Petermann Island. Trathan et al. (2008) reported a decrease in the number of nesting individuals in highly trafficked portions of a colony at Goudier Island (Port Lockroy), positing that younger prospecting birds may preferentially choose unvisited sites. This supports the notion that penguins may habituate to human visitation over time (Holmes et al. 2006; Lynch et al. 2019). More extensive recent work at Goudier Island showed little difference in nest productivity between visited and unvisited portions of the breeding colony (Dunn et al. 2019). Our results, in concert with other recent work on tourism impacts, speak to the efficacy of regulatory measures currently in place for the Antarctic tourism industry and the importance of strict visitation guidelines from organizations such as IAATO. A complete compilation of breeding success estimates, as well as estimates from prior studies is presented in Appendix S1.

## Conclusions

We find no evidence that direct anthropogenic stressors are substantially limiting gentoo penguin breeding success. It may be that climatic events – that is, indirect rather than direct human impacts – are more important determinants of these dynamics. In particular, it seems that extreme snow events are having a disproportionately large impact on chick mortality. This is cause for concern, since an increasing frequency of such events is predicted under climate change scenarios (Uotila et al. 2007). Future work investigating the influence of fine‐scale colony topography (e.g. McDowall and Lynch 2017) on precipitation‐driven nest failure would be a valuable contribution to our understanding of these processes and may provide insights regarding how marginal nesting sites within colonies may relate to a terrestrial carrying capacity. While the impacts of the marine environment on the demographics of the *Pygoscelis* spp. penguins have been given much attention, non‐marine environmental forcing also appears to play a role in the breeding productivity of this species.

This study serves as an important step in camera‐based survivorship methodology and will be important in understanding how other species are responding to these and other stressors. For instance, while gentoo penguin populations are generally stable or increasing in the Antarctic Peninsula region, other *Pygoscelis* spp. (chinstrap and Adélie) penguins are decreasing in abundance in the region. Given the propensity of the chinstrap penguin and Adélie penguin to lay only a single clutch (while the gentoo penguin may re‐lay following a nest failure), extreme climatic events may have a larger demographic impact for these species. These methods may be adapted to a number of species that breed in a stationary location over the course of a breeding season.

The highly non‐linear relationship between precipitation and breeding success illustrates the importance of both intra‐annual dynamics and extreme events in ecological systems and highlights the need for sustained monitoring of these systems. Seasonally averaged breeding success measures (e.g. chicks fledged per nest) do not provide enough information to understand the temporal dynamics of nest failure, and therefore are difficult to interpret with respect to identifying key drivers. Because of the substantial ‘noise’ present, ecologists rely on aggregation to better understand ecological dynamics and the effect of extreme events (Che‐Castaldo et al. 2017). Continuous, high‐resolution monitoring, here facilitated by remote time‐lapse cameras, coupled with appropriate statistical modeling frameworks, such as that presented here, may provide another solution that can help tease apart factors of supposed importance and identification of the impacts of discrete events, such as extreme weather.

## Author Contributions

CY, FMJ, HJL, and TH conceived the study. TH established the camera network. CY and HJL conceived of and performed the statistical analyses. All authors contributed to data annotation. CY and FMJ led the writing of the manuscript. All authors contributed to drafts and gave final approval for publication.

## Supporting information


**Appendix S1.** Image annotation, data availability, model checks, and the impact of precipitation.

**Appendix S2.** Processing and analysis of krill catch data.

**Video S1.** Time‐lapse animation of the impact of a large snow event on chick mortality.

## Data Availability

Data used in this study are available in the Dryad Digital Repository https://doi.org/10.5068/D1MX0Z (Youngflesh et al. 2020).
