# Supplementary material for: Large‐scale assessment of intra‐ and inter‐annual breeding success using a remote camera network
Source: Remote Sens Ecol Conserv. 2020 Aug 31;7(1):97–108. doi: 10.1002/rse2.171 (PMC8048998; doi:10.1002/rse2.171)
Supplement: Supplementary file 1 — Appendix S1. Image annotation, data availability, model checks, and the impact of precipitation. [file RSE2-7-97-s002.pdf]

Appendix S1: Image annotation, data availability, model checks, and the impact of precipitation

Large-scale assessment of intra- and inter-annual breeding success using a remote camera network

Image annotation

Images were captured by Reconyx HC500 Hyperfire Trail Cameras, as part of the *Penguin Watch* remote time-lapse camera network (Fig. S1-1 left). The locations of individual nests were annotated on a representative image from each site/year (Fig. S1-1 middle). The xy coordinates of the nest locations were used to demarcate 'nest zones' through Voronoi tessellation (Fig. S1-1 right). These zones remained constant throughout each site/year, as camera position was fixed.

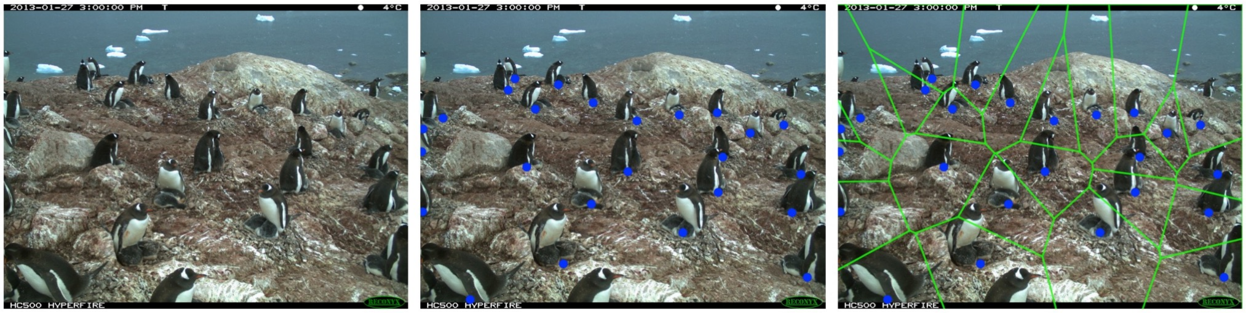

Figure S1-1: Image processing progression - raw image (left) to image with annotated nests (middle) to image with annotated nest zones (right).

Each image captured over the course of the season was uploaded to the Zooniverse platform (Fig. S1-2). We classified the location of penguin chicks in each image obtained from the remote time-lapse camera network from the sighting of the first chick at a site to the crèche stage (the period during which penguin chicks begin to spend less time at the nest and form large chick aggregations). The zones were used as guidelines for users, as chicks from one nest may slightly stray into an adjoining zone. We did not mark any chick where there was any ambiguity regarding which nest it belonged to, as false positives (in the form of incorrect nest membership for a given chick) would bias estimates in this modeling framework. Ambiguous nest membership was rarely an issue in scoring and all classifications were reviewed for accuracy to avoid potential false positives. Chick body position and proximity to nest were used to distinguish live from dead chicks (only live chicks were scored), though there was rarely any ambiguity. This method effectively provides a time series of the number of observed chicks for each nest/site/year (Fig. 1b in main text).

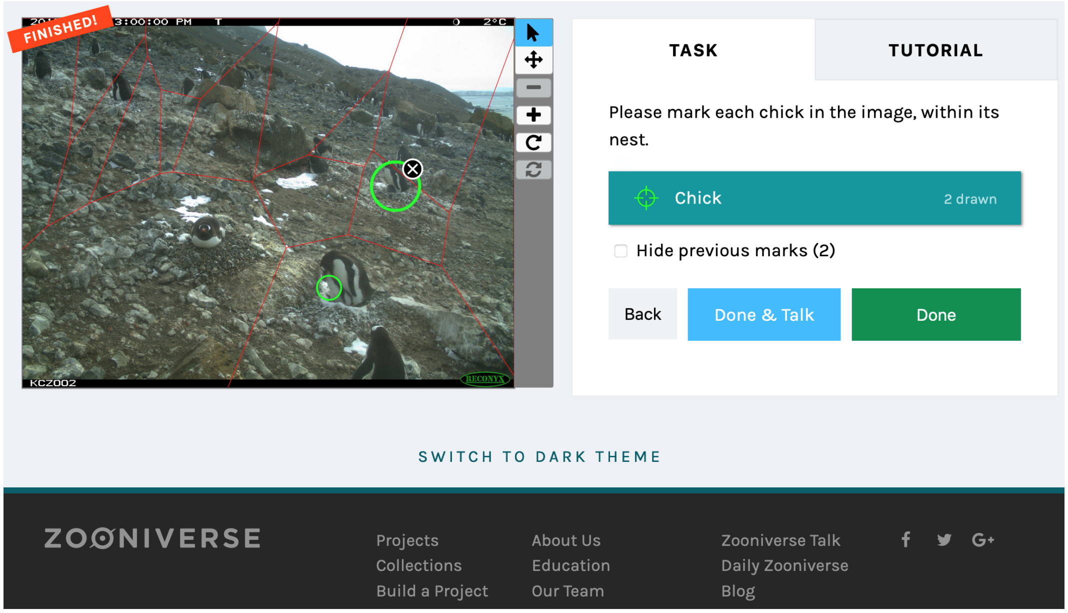

Figure S1-2: Chick classification on the Zooniverse platform. For each image, users classified the location of all visible chicks by clicking on them. The nest zones are represented by the red polygons while user-identified penguin chicks are represented by the green circles.

Data Availability

Table S1-1: Breeding success estimates from this study and estimates compiled from two other studies in the literature (providing an additional 11 site/years of information). Estimates from other studies are provided for reference, though these data were not included in any analyses. Note that measures of uncertainty for estimates from this study (given as posterior standard deviations) are not provided for estimates from other studies in the literature.

Site Name	Year	Latitude	Longitude	Chicks / Pair	Uncertainty (sd)	Source
Brown Bluff	2015-2016	-63.53	-56.89	0.56	< 0.01	This study
Brown Bluff	2017-2018	-63.53	-56.89	1.48	0.07	This study
Cooper Bay	2014-2015	-54.78	-35.83	1.90	< 0.01	This study
Cooper Bay	2016-2017	-54.78	-35.83	1.66	0.05	This study
Cuverville Island	2013-2014	-64.41	-62.38	1.14	< 0.01	This study
Cuverville Island	2014-2015	-64.41	-62.38	1.32	< 0.01	This study
Damoy Point	2013-2014	-64.82	-63.49	1.57	< 0.01	This study
Danco Island	2012-2013	-64.44	-62.37	1.57	< 0.01	This study
Danco Island	2013-2014	-64.44	-62.37	1.31	< 0.01	This study
Danco Island	2014-2015	-64.44	-62.37	1.48	< 0.01	This study
Georges Point	2012-2013	-64.67	-62.67	1.69	< 0.01	This study
Georges Point	2013-2014	-64.67	-62.67	1.50	< 0.01	This study
Georges Point	2014-2015	-64.67	-62.67	1.33	< 0.01	This study
Georges Point	2016-2017	-64.67	-62.67	1.65	< 0.01	This study
Godthul	2017-2018	-54.29	-36.26	1.70	0.03	This study
Jougla Point	2016-2017	-64.50	-63.30	1.53	0.05	This study
Port Lockroy	2012-2013	-64.82	-63.29	1.63	< 0.01	This study
Port Lockroy	2013-2014	-64.82	-63.29	1.33	< 0.01	This study
Port Lockroy	2014-2015	-64.82	-63.29	1.62	< 0.01	This study
Maiviken	2012-2013	-54.24	-36.50	1.62	0.04	This study

Site Name	Year	Latitude	Longitude	Chicks / Pair	Uncertainty (sd)	Source
Mikkelsen Harbor	2016-2017	-62.54	-60.47	1.08	< 0.01	This study
Mikkelsen Harbor	2017-2018	-62.54	-60.47	1.08	0.04	This study
Neko Harbor	2012-2013	-64.86	-62.52	1.87	< 0.01	This study
Neko Harbor	2013-2014	-64.86	-62.52	1.57	< 0.01	This study
Neko Harbor	2014-2015	-64.86	-62.52	1.13	< 0.01	This study
Neko Harbor	2015-2016	-64.86	-62.52	1.57	< 0.01	This study
Ocean Harbor	2014-2015	-54.34	-36.27	1.71	< 0.01	This study
Petermann Island	2014-2015	-65.17	-64.14	1.41	< 0.01	This study
Cape Shirreff	2016-2017	-62.46	-60.79	1.63	-	Hinke et al. 2018
Cierva Cove	2016-2017	-64.14	-60.98	1.47	-	Hinke et al. 2018
Llano Point	2016-2017	-62.18	-58.46	1.53	-	Hinke et al. 2018
Galindez	2016-2017	-65.24	-64.25	1.46	-	Hinke et al. 2018
Lion's Rump	2016-2017	-62.135	-58.13	1.26	-	Hinke et al. 2018
Petermann Island	2016-2017	-65.17	-64.14	1.51	-	Hinke et al. 2018
Petermann Island	2003-2004	-65.17	-64.14	1.52	-	Lynch et al. 2010
Petermann Island	2004-2005	-65.17	-64.14	1.23	-	Lynch et al. 2010
Petermann Island	2005-2006	-65.17	-64.14	1.42	-	Lynch et al. 2010
Petermann Island	2006-2007	-65.17	-64.14	1.46	-	Lynch et al. 2010
Petermann Island	2007-2008	-65.17	-64.14	1.23	-	Lynch et al. 2010

Posterior predictive check - capture-recapture model

Posterior predictive checks were used to assess the degree to which the model could produce data that resemble the observed data. The test statistic chosen was the total number of observed chicks for each site/year. Bayesian p-values represent the proportion of generated datasets with a test statistic (in this case, the total number of chicks 'observed') that is greater than the test statistic derived from the actual data. Bayesian p-values close to zero or one indicate that the model generates data different than those used to fit the model. Results did not indicate any model misfit (as evidenced by Bayesian p-values near 0.5).

Figure S1-3: Posterior predictive check results. Histograms represent the number of chicks 'observed' in the generated data at each iteration of the model for each site/year, while the vertical red lines represent the actual number of chicks observed for each site/year. Bayesian p-values are given for each site/year. BROW - Brown Bluff, COOP - Cooper Bay, CUVE - Cuverville Island, DAMO - Damoy Point, DANC - Danco Island, GEOR - Georges Point, GODH - Godthul, JOUG - Jougla Point, LOCK - Port Lockroy, MAIV - Maiviken, MIKK - Mikkelsen Harbor, NEKO - Neko Harbor, OCEA - Ocean Harbor, PETE - Petermann Island

Precipitation coding

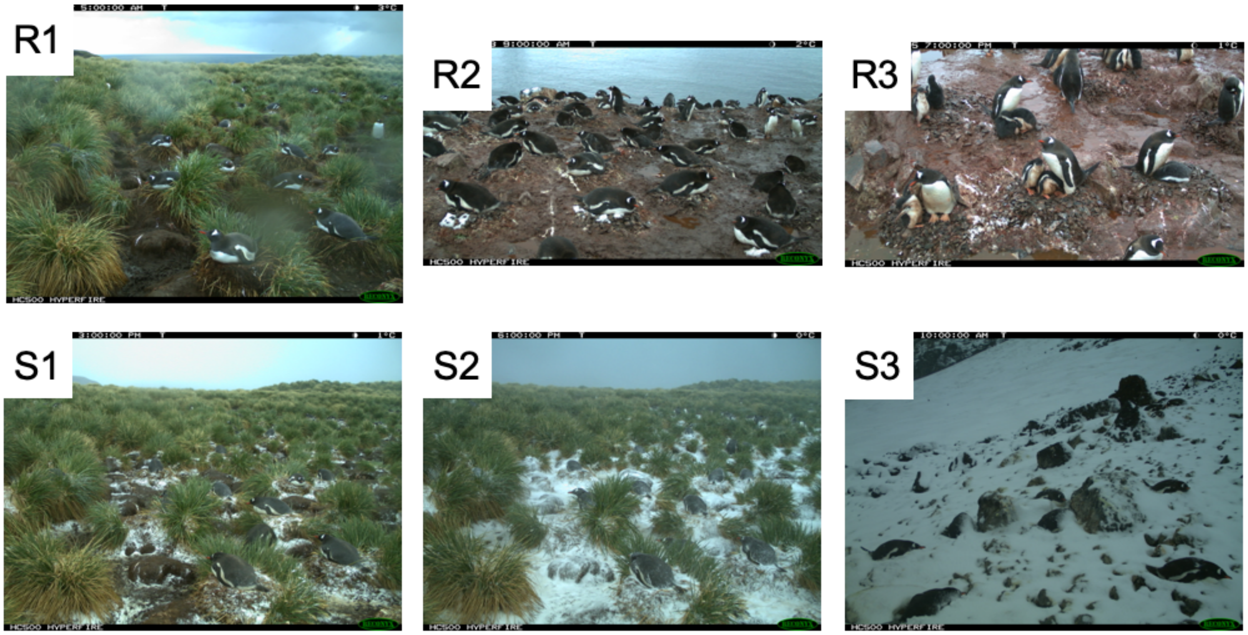

Figure S1-4: Example images used for precipitation coding. The top row represents rain events, while the bottom row represents snow events. For each precipitation type, the magnitude of the events increases from left to right (R1/S1 left, R2/S2 middle, R3/S3 right).

Effect of precipitation events on the timing of nest failure

While smaller precipitation events were of little consequence for chick mortality (Fig S1-5), large precipitation events appear to impact breeding success in a non-linear fashion (Fig. 5, Video S1). Extreme weather events can result in increased egg failure or chick mortality.

For the plots below, mean detection probability at each site in every year at every time step $\overline{p_{t,j,k}}$ was calculated as a derived quantity by averaging across the detection probabilities $p_{t,i,j,k}$ for all nests i,

$$\overline{p_{t,j,k}} = \frac{1}{N_{j,k}} \sum_{i} p_{t,i,j,k}$$

where N is the number of nests for a given site and year.

Figure S1-5: Number of chicks alive at each time point in the season for each site/year. The blue dashed lined represents the transition from the extrapolated egg lay date to the date the first penguin chick was recorded in an image. The solid blue line is the latent state Z, the total number of chicks at a given site/year at any given time point. The blue ribbon represents one standard deviation around Z. The red line is the mean detection probability at each time step across all nests for that site $\bar{p}$. The red ribbon represents one standard deviation around $\bar{p}$ (though the ribbon is not visible in most cases due to the small degree of uncertainty for $\bar{p}$). Snow and rain events are represented by purple and orange vertical lines, respectively. BROW - Brown Bluff, COOP - Cooper Bay, CUVE - Cuverville Island, DAMO - Damoy Point, DANC - Danco Island, GEOR - Georges Point, GODH - Godthul, JOUG - Jougla Point, LOCK - Port Lockroy, MAIV - Maiviken, MIKK - Mikkelsen Harbor, NEKO - Neko Harbor, OCEA - Ocean Harbor, PETE - Petermann Island

Alternative visualization of changes in chicks over time

Figure S1-6: An alternative visualization (to Fig S1-5) of the number of chicks alive at each time point in the season for each site/year. The blue dashed lined represents the transition from the extrapolated egg lay date to the date the first penguin chick was recorded in an image. Each solid blue line represents one realization (i.e. iteration of the posterior chain) of the latent state Z, the total number of chicks at a given site/year at any given time point. A total of 500 realizations are displayed. The red line is the mean detection probability at each time step across all nests for that site $\bar{p}$. The red ribbon represents one standard deviation around $\bar{p}$ (though the ribbon is not visible in most cases due to the small degree of uncertainty for $\bar{p}$). BROW - Brown Bluff, COOP - Cooper Bay, CUVE - Cuverville Island, DAMO - Damoy Point, DANC - Danco Island, GEOR - Georges Point, GODH - Godthul, JOUG - Jougla Point, LOCK - Port Lockroy, MAIV - Maiviken, MIKK - Mikkelsen Harbor, NEKO - Neko Harbor, OCEA - Ocean Harbor, PETE - Petermann Island
