# Supplementary material for: Large‐scale assessment of intra‐ and inter‐annual breeding success using a remote camera network
Source: Remote Sens Ecol Conserv. 2020 Aug 31;7(1):97–108. doi: 10.1002/rse2.171 (PMC8048998; doi:10.1002/rse2.171)
Supplement: Supplementary file 2 — Appendix S2. Processing and analysis of krill catch data. [file RSE2-7-97-s001.pdf]

Appendix S2: Processing and analysis of krill catch data

Large-scale assessment of intra- and inter-annual breeding success using a remote camera network

Commission for the Conservation of Antarctic Marine Living Resources (CCAMLR) Small-Scale Management Units (SSMUs)

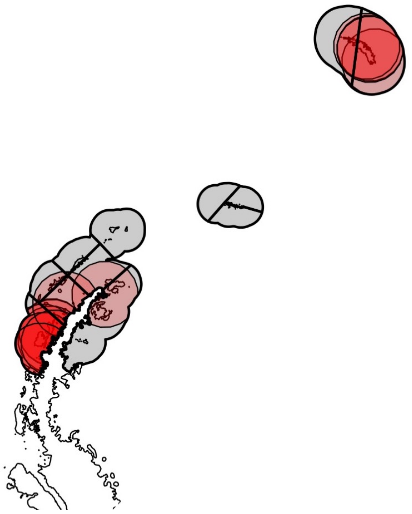

Figure S2-1: Commission for the Conservation of Antarctic Marine Living Resources (CCAMLR) Small-Scale Management Units (SSMUs) are represented as grey shaded regions. The 150 km buffers around each site used in this study are represented by red circles.

Effect of krill fishing on penguin breeding success during the breeding season

Analyses presented in the main text used the sum of the weighted average krill catch from March (of year t-1) through January (year t), representing all krill caught at a site prior to and during the breeding season. Krill catch over just the breeding season (December - January) was also calculated using a 25km buffer to test for the effect of krill extraction during the breeding season in areas where gentoo penguins may be foraging during this time period.

Breeding success was modeled as a function of krill catch during the breeding season,

$$bs_{obs_i} \sim N(bs_{true_i}, \sigma_{bs_i})$$

$$bs_{true_i} \sim N(\mu_i, \sigma)$$

$$\mu_i = \alpha + \beta \times x_i$$

where bs_{obs} is the estimated breeding success (posterior mean of the derived quantity from the capture-recapture model) for a given site/year, σ_{obs} is the uncertainty about the estimate of breeding success for each site/year (posterior standard deviation of the derived quantity; this is given rather than estimated by the model), bs_{true} is the latent true state of breeding success, x is the covariate of interest for site/year i, α is the intercept parameter, β is the slope parameter, and σ is the residual error term.

Models were fit using the R package 'rjags' to interface with JAGS in the R statistical environment. Inferences were obtained from 10,000 samples drawn from four chains, following a 'burn-in' period of 10,000 draws and

an adaptation phase of 5000 draws. All chains unambiguously converged (Rhat < 1.1) and effective samples sizes were sufficiently large (ess / chain > 100). Results are similar to those presented in the main text, showing no clear relationship between breeding success and krill catch.

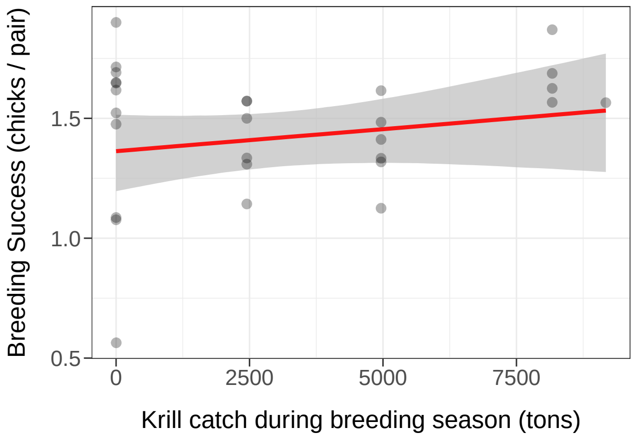

Figure S2-2: Breeding success as a function of krill catch during the penguin breeding season. The red line represents the posterior median. The gray ribbon represents the 95% credible interval. Median slope (β) = 1.8×10^{-5} [95% CI: -1.8×10^{-5} - 5.4×10^{-5}].

Temporal distribution of krill fishing effort

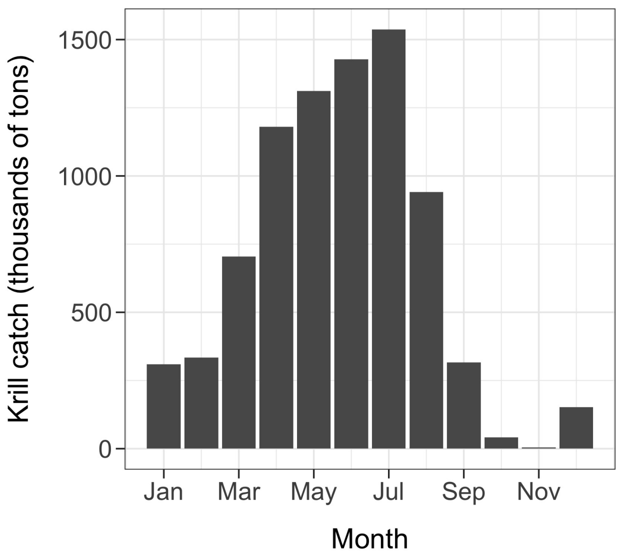

Figure S2-3: Total monthly reported krill catch over the course of the calendar year for all sites in this study. Monthly totals were averaged across all focal years (2013-2018). Krill fishing at these sites (within the 150km buffers) is highest during the overwinter period.
